# Oxidative Stability and Antioxidant Activity of Selected Cold-Pressed Oils and Oils Mixtures

**DOI:** 10.3390/foods11111597

**Published:** 2022-05-28

**Authors:** Edyta Symoniuk, Małgorzata Wroniak, Katarzyna Napiórkowska, Rita Brzezińska, Katarzyna Ratusz

**Affiliations:** 1 Department of Food Technology and Assessment, Institute of Food Science, Warsaw University of Life Sciences, Nowoursynowska St. 159c, 02-787 Warsaw, Poland; malgorzata_wroniak@sggw.edu.pl (M.W.); katarzyna.krosnicka@vp.pl (K.N.); katarzyna_ratusz@sggw.edu.pl (K.R.); 2 Department of Chemistry, Institute of Food Sciences, Warsaw University of Life Sciences, Nowoursynowska St. 159c, 02-787 Warsaw, Poland; rita_brzezinska@sggw.edu.pl

**Keywords:** ABTS, antioxidants activity, DPPH, cold-pressed oils, oxidative stability, Rancimat

## Abstract

The aim of the study was to analyse the chemical composition and oxidation stability of selected cold-pressed oils and oil mixtures. The oils were tested for their initial quality, fatty acid composition, total phenolic compounds, DPPH, and ABTS free radical scavenging activity. The Rancimat method was used to assess oxidative stability. The obtained results were subjected to principal component analysis (PCA) to determine the influence of selected chemical properties on the oxidative stability of the oil. It has been found that different factors of oil quality influence the stability of cold-pressed oils. The highest correlation coefficient was noted between the induction time, peroxide value, and TOTOX indicator (r = 0.89). Fatty acid composition, including the percentage of SFA, MUFA, PUFA, and the ability to scavenge ABTS captions radicals, did not significantly affect the oxidative stability of the oils. Black cumin seed oil was the most resistant to the oxidation processes in the Rancimat apparatus, mainly due to the high content of phenolic compounds (384.66 mg GAE/100 g). On the other hand, linseed oil and its mixtures were the least stable. Their fatty acid composition was dominated by a polyunsaturated α-linolenic fatty acid, significantly reducing the antioxidant resistance.

## 1. Introduction

Pressing is one of the oldest and most popular methods used to extract oil from seeds and fruits. Currently, two methods of pressing can be distinguished due to the temperature used during the process. One of them is cold pressing, where the oil temperature should not exceed 50 °C. The oil obtained in this way can only be cleaned by means of sedimentation, centrifugation, and filtration. The second method is hot pressing with process temperatures often above 100 °C. In this case, the oil is subjected to chemical refining due to the presence of many impurities dissolved in the oil (phosphorus compounds, chlorophylls, metals, free fatty acids, sulphur compounds) [1].

The cold pressing technology is based solely on a mechanical process, without the interference of chemical solvents and high temperatures. As a result of this, it is considered a simple, cheap, and above all, ecological method [2]. It is difficult to obtain consistent product quality in cold-pressed oils. The quality of cold-pressed oils largely depends on the raw material, including its purity, maturity, homogeneity, and having no damage. The deadline, harvesting method, and storage conditions are also important [3].

The diverse composition of cold-pressed oils significantly impacts their durability and the oxidation process. Oxidation is an important process that negatively affects the quality of cold-pressed oils. First of all, it leads to a loss of nutritional value. The oxidation process contributes to the degradation of bioactive compounds beneficial for the human body and the formation of anti-nutritional compounds, even of a carcinogenic nature. In addition, oxidation affects the sensory qualities of the oil, significantly worsening its taste and smell. Among edible fats, cold-pressed oils with a high content of polyunsaturated fatty acid are particularly susceptible to oxidation processes [4,5].

Oxidative stability is one of the most critical features of the quality and durability of edible oils. It depends on the composition of fatty acids, mainly unsaturated fatty acids. Furthermore, the content of antioxidants, primary and secondary oxidation products, or oil impurities is also significant. They are found in greater amounts in hot-pressed oil than in cold-pressed or refined because of the use of high temperatures in this process. This indicator allows for determining the resistance of a given oil to the oxidation processes, which significantly reduces the fats’ quality. Temperature is also a factor determining oxidative stability, so the oxidation induction time of oils shortens with increasing temperature [6,7].

Accelerated methods, which are easy and do not require reagents, are commonly used to assess the stability of oils. However, the oxidation process in such methods takes place at high temperatures and has a different course than at ambient temperature. Determining the stability of oils for different temperatures allows for determining the parameters of the oxidation kinetics, which help estimate the durability of the oil. Our previous works focused on the influence of individual quality parameters on the stability of different cold-pressed oils [4,8]. This issue was raised by other authors; Mikołajczyk and Tańska [9] investigated the influence of the composition on oxidative stability for linseed oil, and Nederal et al. [10] for pumpkin oil. Despite there being scientific reports aimed at assessing the influence of the chemical composition of the oil on its stability, there is no clear statement of what has the greatest impact on the stability of cold-pressed oils. This is probably due to a very large variety of oils in terms of chemical composition and dependence of the quality of the oil on the quality of the raw material, as well as the method of production. Therefore, it is very important to continue research related to the problem of oil stability.

Consequently, it was decided to test various cold-pressing oils and their blends in terms of chemical composition and oxidation stability, with particular emphasis on the parameters of oxidation kinetics. According to the present state of our knowledge, there is no such research. So far, the dependence between oil composition and its oxidation kinetics parameters has not been investigated.

## 2. Materials and Methods

### 2.1. Research Material

The research material consisted of eleven commercial cold-pressed oils. Eight of them were derived from different oilseeds, and three were mixtures. The following oils were analysed: pumpkin (PO), hemp (HO), linseed (LO), camelina (CO), rapeseed (RO), black cumin (BCO), milk thistle (MTO), evening primrose (EPO), and mixture 1 (M1—70% LO, 27% MTO, 3% EPO), mixture 2 (M2—80% RO, 17% LO, 3% EPO), and mixture 3 (M3—91% LO, 5% EPO, 4% PO). The research material was packaged in dark glass bottles with a capacity of 0.25 L. All oils came from the same Polish producer. The oils were delivered directly after the pressing process, at the initial use-by date. The tests were carried out over two weeks, and the oils were stored in freezing conditions (temperature −18 °C) during this time.

### 2.2. Oils’ Initial Quality Assessment

The cold-pressed rapeseed oils were analysed for peroxide value (PV), *p*-anisidine value (*p*-AnV), and K232 and K268 extinction coefficients, which were measured following EN ISO standard methods [11,12,13]. The total oxidation value (TOTOX indicator) was calculated as TOTOX indicator = (2 × PV) + *p*-AnV.

### 2.3. Determination of the Fatty Acid Profile

The fatty acid composition of cold-pressed oils was performed following the AOAC method [14] using gas chromatography. The methyl esters of the test cold-pressed oils were prepared by dissolving 0.1 g of the oil in 6 cm^3^ of hexane and 0.5 cm^3^ of 2 M methanolic KOH. The sample prepared in this way was thoroughly mixed and left to stand until phase separation. Then 1 cm^3^ of the hexane phase was placed in a vial. A ThermoScientific Trace 1300 gas chromatograph with an FID flame ionisation detector was used to determine the fatty acid profile. An SGE BPX70 high-polar capillary column was used to separate the fatty acid esters. The temperature in the first four minutes was 100 °C and raised to 240 °C (at a rate of 3 °C/min). The carrier gas was helium with a flow of 40 cm^3^/min. The standard mixture of 37 FAME methyl esters from Restek was used to identify the fatty acids. The results were reported as the weight percent of the total fatty acid content.

### 2.4. Total Phenolic Content Determination

The total content of phenols in the cold-pressed oils was determined using the Folin–Ciocalteu (F–C) reagent, according to the method described by Mińkowski et al. [15]. A sample of the oil (3 g) was dissolved in 15 cm^3^ of hexane and was extracted three times with methanol (3 × 5 cm^3^) by vortexing. After stratification, the methanol fraction was washed with hexane to remove residual oil. Then, 2 cm^3^ of extract was taken and transferred with 0.5 cm^3^ of the Folin–Ciocalteu reagent to 10 cm^3^ flasks. The mixture was shaken and allowed to stand for 3 min, then 1 cm^3^ of a saturated sodium carbonate solution was added and made up to 10 cm^3^ with demineralised water. After one hour, the absorbance of the samples was measured at a wavelength of λ = 725 nm. The results of the quantitative determinations are presented as gallic acid equivalent (GAE). For this purpose, a calibration curve was prepared in the concentration range from 0.1 to 0.5 mg GAE/mL.

### 2.5. Determination of the Total Antioxidant Capacity by the Reduction in DPPH Free Radical

The determination of the total antioxidant capacity using DPPH free radicals was performed, according to the methodology described by Espin et al. [16]. An amount of 3 cm^3^ of methanol and 1 mL of DPPH were added to 1 cm^3^ of methanolic oil extract. The absorbance of the solution was measured at the wavelength λ = 517 nm. Measurements were made every 2–3 min in two parallel repetitions for an hour.

The exact concentration of DPPH in the reaction medium was determined from a calibration curve prepared with standard Trolox solutions (TEAC) in the concentration range of 0 to 50 mM/kg.

### 2.6. Determination of Total Antioxidant Capacity by ABTS Radical Cation Reduction Method

The total antioxidant capacity was determined using ABTS cation radicals (3-ethylbenzothiazoline 6-sulfonic acid) according to the methodology described by Czeniakowska-Szydło and Łaszewska [17]. The first stage of the determination was the preparation of radical cation by dissolving ABTS and potassium persulfate in methanol, and absorbance at the wavelength λ = 734 nm was equal to 700. The fully prepared, tightly closed solution was stored in a dark room for 24 h. Then, 2.5 cm^3^ of methanol extract was added to 110 µL of prepared cation radicals. The absorbance of the solution was measured at the wavelength λ = 734 nm. Measurements were made every 2–3 min in two parallel repetitions for an hour. The exact concentration of ABTS in the reaction medium was determined from a calibration curve prepared with standard Trolox solutions (TEAC) in the concentration range of 0 to 50 mM/kg.

### 2.7. Determination of Oxidative Stability in the Rancimat Apparatus

The oxidation stability measurement was carried out following EN ISO 6886:2009 [18] in the Rancimat type 892 apparatus by Metrohm (Switzerland). Oil samples weighing 2.5 g were placed in a heating block, then a stream of air of 20 L/h was passed through the sample. The volatile oxidation products formed during the measurement were transferred along with the air stream to the measuring vessel containing 60 cm^3^ of deionised water. Measurement was carried out at five temperatures: 90, 95, 100, 105, and 110 °C for hemp and linseed oil and 100, 105, 110, 115, and 120 °C for other oils. The determination of oxidative stability for each of the tested oils was performed in two parallel repetitions, of which their arithmetic mean was given as the final result.

### 2.8. Determination of the Parameters of Oxidation Kinetics Using the Rancimat Method

In order to determine the parameters of the oxidation kinetics, the procedure described by Kowalski et al. [19] was followed. The oxidation induction times obtained during the determination of the oxidative stability with the Rancimat test were used for the calculations. Based on the obtained results, graphs were prepared on a semi-logarithmic scale, the dependence of the decimal logarithm of the induction time on temperature and the reciprocal temperature.

The regression lines were determined according to the following equation:

τRancimat = 1/T,(1)

where: τRancimat—induction time determined in the Rancimat, T—oxidation temperature [°C].

Using the obtained results and the Arrhenius formula:

*k* = Zexp (−Ea/RT), (2)

The basic parameters of the oil oxidation kinetics were calculated: activation energy (Ea), pre-exponential factor (Z), and reaction rate coefficient (*k*) at measurement temperatures.

The enthalpy (ΔH) and entropy (ΔS) were calculated based on the equation derived from the activated complex theory:

ln(k/T) = ln(k_B_/h) + (ΔS/R) − (ΔH/RT), (3)

where: k_B_—Boltzmann constant (1.380649 × 10^−23^ J/K), h—Planck’s constant (6.6260755 × 10^−34^ Js), R—gas constant (8.314 J/mol K).

### 2.9. Statistical Analysis of the Results

All experiments were carried out in triplicate. Statistica version 13.3 (StatSoft, Inc., Tulsa, OK, USA) was used to analyse the obtained experimental results. One-way analysis of variance (ANOVA) and Tukey’s test were performed with a *p*-value ≤ 0.05. Additionally, principal component analysis (PCA) was performed. The results of the cluster analysis are presented graphically using a hierarchical breakdown. The chart shows the degree of influence of individual variables on the main components. The Pearson correlation coefficient distribution table was used to determine the significance of the correlations obtained in the PCA test.

## 3. Results and Discussion

### 3.1. Oils Oxidation Indices

Results of the oils oxidation indices are summarised in Table 1. The PV fluctuated at the level of 0.33–81.93 mEq O_2_/kg. BCO had the highest level of primary oxidation products. Its PV was more than five times higher than the maximum specified in the Codex Alimentarius [20]. Ying et al. [21] also obtained a high peroxide value for black cumin oil (57.72 mEq O_2_/kg). The amount of primary oxidation products is influenced by the period and method of oil storage. However, in the case of black cumin seed oil, the overestimated values may also result from the specific nature of the raw material. This oil contains significant amounts of volatile essential oils, so the method used for this determination may be inappropriate, and the results are unreliable [21,22,23]. PO (42.52 mEq O_2_/kg) and HO (19.42 mEq O_2_/kg) were also characterised by an excessively high PV. Ying et al. [21] for hemp oil determined a much lower content of primary oxidation products, amounting to 10.96 mEq O_2_/kg. When analysing the obtained results, it should be taken into account that the tested oils were commercial products. Therefore, the conditions of their production and transport were unknown. Moreover, we do not know the quality of the seeds used for pressing. We have no knowledge of their maturity, humidity, damage, and storage conditions. Quality control of the raw material is essential as the quality of oil from unsuitable seeds cannot be improved. Thus, the high content of peroxides may prove a significant degree of oxidation of individual oils before starting the research. The remaining oils met the requirements of the standard. The lowest PV was for linseed, rapeseed, and milk thistle oil, and their values ranged from 0.33 to 0.95 mEq O_2_/kg, respectively.

The *p*-anisidine value of the tested oils ranged from 0.49 to 7.14. In RO, MTO, M1, and M2 oils the *p*-AnV values were higher than the PV, proving advanced transformations of primary oxidation products. PO was characterised by the highest level of secondary oxidation products—7.14. Slightly higher values were recorded by Kurzeja et al. [24] when analysing the oxidative stability of cold-pressed oils, where the *p*-AnV after pressing was 8.09. LO had the lowest level of secondary oxidation products (0.49). The obtained values were similar to the results published by other authors. The *p*-AnV given by Choo et al. [25] and Symoniuk et al. [4] ranged from 0.07 to 1.43. In the case of linseed oil mixtures, the content of secondary oxidation products was much higher and amounted for M3—2.59, M2—2.76, M1—5.51.

Calculated TOTOX index for the tested cold-pressed oils ranged from 2.38 for LO to 167.76 for BCO. According to Bojanowska and Lamarska [26], the borderline level determining good oil quality is TOTOX, equal to 10. Among the analysed oils, half of them exceeded this limit. BCO achieved the highest TOTOX index due to the high PV and *p*-AnV values. Similarly, PO with a TOTOX of 92.18. The lowest fat oxidation index was found for LO (2.38), MTO (2.98), M2 (3.29), RO (5.25), and M1 (6.16). A similar result for cold-pressed rapeseed oil was obtained by Kruszewski et al. [27] 4.46–8.31. The obtained value of 2.38 was significantly lower than the values obtained by Symoniuk et al. [28], where the general degree of oxidation of commercial linseed oils ranged from 3.11 to 9.07.

### 3.2. Specific Extinction Coefficient under UV Light

Table 1 shows the results of K232 and K268 coefficients of the tested cold-pressed oils. The K232 extinction coefficient, informing about the content of conjugated fatty acid dienes and primary oxidation products, was from 0.26 to 5.61. On the other hand, the K268 coefficient values, defining the level of conjugated trienes and secondary oxidation products, were much lower and oscillated between 0.03 and 3.63. MTO had the lowest K232 (0.26) and K268 (0.03) extinction values. For RO, the obtained results were slightly higher and differed significantly from MTO, the K232 coefficient was 1.45 and the K268 was 0.17. Rabiej-Kozioł et al. [29] obtained significantly higher results for both determinations. The cold-pressed rapeseed oil tested by them had values of 2.775 (K232) and 0.522 (K268). BCO (5.61) and PO (3.63) had a high level of conjugated diene (K232).

Currently, there is no standardised limit value for K232 and K268 for cold-pressed oils. However, when discussing the results, it is worth referring to the EU Commission Regulation 2568/91 [30] for extra virgin olive oil. According to the Regulation, this value for K232 should not be higher than 2.50, and the value for K268 should not be higher than 0.22. Regarding the content of conjugated trienes (K268), only MTO and RO did not exceed the limit value laid down in the EU Regulation. The remaining analysed oils were characterised by significantly higher values of the K268 extinction coefficient, ranging from 0.23 (M2) to 3.63 (PO). 

### 3.3. Fatty Acid Composition of Analysed Cold-Pressed Oils

The fatty acid composition is one of the most important determinants of oil quality. According to the obtained results (Table 2), all oils were characterised by their typically fatty acid compositions [4,26,28]. According to the data in the literature, the composition of fatty acids significantly influences the stability of oils. The rate of C18:2 acid oxidation is 10–40 times higher than that of C18:1, and the rate of C18:3 oxidation is 2–4 times faster than that of C18:2 [31]. Among all oils, HO, LO, and M2 had the highest content of unsaturated fatty acids. The highest α-linolenic acid content was found in linseed, camelina oil, and M2. Another polyunsaturated fatty acid, linolenic acid, was most abundant in EPO, BCO, HO, and PO. The highest amount of monounsaturated acids was present in the rapeseed oil; this oil also had the lowest content of saturated fatty acids.

### 3.4. Total Phenolics Content of Analyzed Cold-Pressed Oils

Phenols are a large and quite diverse group of compounds. They can occur in the form of phenolic acids, lignans, flavonoids, alcohols, and oleuropein derivatives [32]. Phenolic compounds are characterised by strong antioxidant properties, especially anti-free radicals [15,33]. Phenolic compounds are native antioxidants and, to a large extent, inhibit the oxidation processes taking place in oil. They are present only in cold-pressed oils, as they are completely removed in the refining process during the neutralisation stage. TPC was determined using the Folin–Ciocalteu (F–C) method in the extracts of selected cold-pressed oils. This method is widely used because it enables the determination of all phenols, regardless of their structure [34]. The obtained results are expressed as gallic acid equivalents and are presented in Table 3.

The analysis of the obtained results showed that cold-pressed oils from individual raw materials are characterised by a very diverse content of phenolic compounds. The TPC amount in the analysed oils ranged from 56.61 (LO) to 384.66 (BCO) mg of gallic acid per 100 g of oil. BCO (384.66 mg GAE/100 g) was distinguished by the highest content of phenols among all the tested oils. A slightly lower value of 310.26 mg GAE/100 g was published by Sultan et al. [35]. BCO oil was also researched by Haron et al. [36]. Their results were wildly divergent due to the origin of the raw material (Iran, Malaysia, Yemen) and ranged from 96 to 760 mg GAE/100 g. The determined TPC of black cumin oil rich in essential oils could result from the method’s specifics. According to Padd and Pich [37], the Folin–Ciocalteu reagent can react with easily oxidisable substances that are not considered phenolic compounds, thereby overestimating the total phenolic content. The total content of phenolic compounds in the oil depends on many factors, including the quality of the raw material, the climatic conditions of cultivation, the method of production, packaging, or storage [38]. A relatively good source of phenolic compounds was MTO (252.87 mg GAE/100 g) and HO (236.25 mg GAE/100 g). Yu et al. [39] for hemp oil presented the total content of phenols as an equivalent of gallic acid at the level of 44 mg/100 g. Siger et al. [38] obtained much lower results in their work, where the content of phenolic compounds expressed as an equivalent of caffeic acid was 2.45 mg/100 g oil. The differences in the presented results were influenced by the extraction method, solvent, and different acids for the standard curve. Nevertheless, in the research of Siger et al. [40], hemp oil also had the highest content of phenolic compounds among all the oils tested (SO, RO, CO, PO). In the remaining tested cold-pressed oils, the total phenol content was much lower and ranged from 56.61 to 162.43 mg GAE/100 g. The mixture with the lowest linseed oil content (M1) had the highest amount of phenolic compounds (125.27 mg GAE/100 g). It did not differ significantly from evening primrose oil or camelina oil. Moreover, very divergent values for the total value of phenolic compounds in linseed oil are published in the literature. Mińkowski et al. [15] report that the total value of phenolic compounds in linseed oil is 1.17 mg FAE/100 g, and Prescha et al. reported [41] 1.19 mg/100 g. Linseed oils were tested by Choo et al. [25], and their total content in terms of ferulic acid ranged from 76.8 to 307.3 mg/100 g. Symoniuk et al. [4], using the same acid for the determination, obtained results from 60.25 to 115.12 mg FAE/100. The presented differences result from the type of extractant used, the variety, or the raw material’s overall quality. CO contained more phenolic compounds (132.67 mg GAE/100 g) than linseed oil (56.61 mg GAE/100 g). Abramovič et al. [42], examining the camelina oil, obtained a similar result of 128 mg GAE/100 g.

### 3.5. Antioxidant Capacity Determined by the Reduction in DPPH Free Radicals and ABTS Cation Radicals

Oil’s antioxidant properties can be determined by deactivating free radicals that activate the oxidation processes occurring in fats. In cold-pressed oils, free radicals react mainly with phenolic compounds, carotenoids, tocopherols, tocotrienols, and some sterols [43]. The antioxidant activity of the tested cold-pressed oils was determined using 2,2-diphenyl-1-picrylhydrazyl (DPPH) free radicals and 3-ethylbenzothiazoline 6-sulfonic acid (ABTS) radicals. The obtained results are summarised in Table 3. The antioxidant capacity measured using DPPH free radicals ranged from 2.41 to 3.65 mM TEAC/kg. EPO (3.65 mM TEAC/kg) was characterised by the highest activity of DPPH radical neutralisation. The research of Symoniuk et al. [4] showed much lower values (0.55–0.87 mM TEAC/kg) and reported that evening primrose oil, among all the oils tested (linseed, rapeseed, bush oil, hemp), was characterised by the weakest antiradical properties. These differences may result from the specificity of the raw material, its variety, or cultivation conditions [4]. MTO (2.59 mM TEAC/kg) showed a moderate ability to scavenge free radicals. Similar results were obtained by Prescha et al. [41] and Symoniuk et al. [4]. The cited authors determined the antioxidant capacity at the level of 2.14–2.56 mM TEAC/kg of oil. Although BCO had the highest content of phenolic compounds, its ability to radically inactivate free radicals was the lowest at 2.41 mM TEAC/kg. Górnaś et al. [44], examining soybean oil, also found that oil with a high content of phenols does not always show the best antiradical activity. The type, amount, and relative proportions of antioxidants and the presence of accompanying substances in the oil may have an impact on the effectiveness in combating free radicals. It should also be emphasised that the overall content of phenolic compounds and the chemical composition of the compared oils were varied, which also influenced the demonstrated antioxidant activity [45]. The results of the determined antioxidant activity with the use of the radical ABTS (Table 3) ranged from 3.87 (MTO) to 11.62 mM TEAC/kg (BCO). The values obtained were 7.53 and 5.73 mM TEAC/kg oil, respectively. Szydłowska-Czerniak and Tułodziecka [46] also investigated the antioxidant capacity of methanol rapeseed extracts using ABTS cation radicals. The authors determined the antioxidant capacity of oilseed rape seed extract in winter at the level of 22.48 and spring at 26.73 mM TEAC/100 g. Based on the statistical analysis, it can be concluded that the rapeseed oil extract showed a much greater antioxidant capacity than its 80% mixture (M2).

Based on the obtained results, it can be concluded that the tested cold-pressed oils had higher antioxidant activity in the determination with the use of DPPH free radicals than ABTS. It proves the higher activity of lipophilic antioxidants (tocopherols) than hydrophilic antioxidants (phenolic compounds), which was also confirmed in the research by Espin et al. [16] and Prescha et al. [41]. The recorded values differ because the DPPH method applies to both the hydrophilic and lipophilic fractions, and the ABTS only applies to the hydrophilic fraction [47]. The potential ability of the tested samples to counteract the oxidation reaction was also expressed as a percentage of free radical scavenging (% inhibition). A high percentage of inhibition indicated the strong antioxidant properties of the compound and a slight residue of unreacted radicals [48].

BCO (44.69%) was characterised by the lowest ability to scavenge DPPH free radicals, and the time of which taken for the radical concentration reduction exceeded the measurement time by 50% (>60 min). Slightly lower results for this assay were obtained by Sultan et al. [33]—32.32%. On the other hand, Pawłowska et al. [49] noted a much higher value of 75% for black cumin oil. They also found a strong correlation between the ability to inhibit DPPH radicals and the content of primary oxidation products. The difference may be the chemical composition, including the phenol content and the origin of the raw material. EPO had the highest capacity for scavenging DPPH free radicals, with 88.47% inhibition. This oil also had the shortest TEC50 time. The concentration of free radicals was halved after only 1.85 min. However, many authors report that olive oil has an even greater ability to scavenge radicals. Kirlan et al. [50] reported that olive oil is able to reduce 52.31–94.91% of DPPH radicals. In the case of the method with ABTS radicals, the tested cold-pressed oils showed significant differences in the ability to deactivate ABTS radicals. The radical cation scavenging ability ranged from 36.53 to 92.55%. BCO was characterised by the highest ability to capture radical cation, the inhibition of which was 92.55%, and the time of reduction in the radical concentration by 50% was only 0.05 min. The differences in the results with the determination of DPPH free radicals and ABTS cation radicals indicate that BCO has many more phenolic compounds classified as hydrophilic antioxidants than tocopherols, which are lipophilic antioxidants. In turn, the lowest inhibition value was found in MTO. In the case of MTO, TPC results did not affect the cation scavenging ability. A given assay, which had a relatively high amount of phenols (252.87 mg GDA/100 g), showed the lowest inhibition. In many research studies, the authors confirm this relationship and suggest that the ability to scavenge free radicals depends primarily on the determination method and does not always correlate with the content of phenolic compounds [51].

### 3.6. Oxidative Stability in the Rancimat Apparatus

Oxidative stability is one of the most important features of the quality and durability of cold-pressed oils. This indicator allows you to determine the resistance of a given oil to the oxidation processes that significantly reduce the quality of fats. Oxidative stability depends on the composition of fatty acids, especially unsaturated ones, which are susceptible to oxidation. The content of antioxidants, primary and secondary oxidation products, or oil impurities is also important [6,7]. Temperature also determines oxidative stability because the oils’ oxidation induction time decreases with increasing temperature.

The results of the Rancimat method at 100 °C (Table 4) show that the oils with the lowest oxidation stability were LO (3.37 h) and its mixes, also HO (4.32 h) and BCO (4.62 h). These oils did not differ statistically significantly. BCO turned out to be the most stable among the oils tested, with an induction time of 38.34 h. The composition of fatty acids largely determined the oxidation rate of oils. Due to the highest content of α-linolenic acid (53.90%), LO was characterised by the lowest oxidative stability, and its induction time was only 3.37 h. The M1 had an equally large amount of this acid (56.03%), the induction time was 3.47 h, and it did not differ significantly from LO.

The result obtained in a given experiment was similar to the data in the literature. The induction time of the oil tested by Marszałkiewicz et al. [52] ranged from 4.79 to 7.51 h. According to the researchers, the stability of the oil was also influenced by its peroxide value. The results obtained in the study also confirm this relationship. The analysed oils with an initial low PV were more susceptible to oxidation than those with a significant value of primary oxidation products. CO had an equally low oxidative stability (4.62 h). M2 in the Rancimat test had an oxidation induction time of 1.87 h shorter than that of RO without other oils. The reduction in oxidative stability in the rapeseed oil mix was caused by the addition of 17% linseed oil. The high content of PUFA (Table 2) is very unstable and probably accelerated the oxidation process. The induction time for rapeseed oil was 15.45 h. Much lower values for this oil were obtained by Rękas et al. [53] (4.08 h). The shorter induction time resulted from the use of a higher process temperature (120 °C) by the authors. According to the van’t Hoff rule, an increase in temperature by 10 K causes a 2–4-fold increase in the reaction rate. Therefore, the time of formation of oxidation products at 100 °C was much longer. PO was characterised by a relatively long induction period–22.45 h. The stability of cold-pressed pumpkin oil was also investigated by Neđeral et al. [10]. Their values differed slightly from those obtained in this study and amounted to 24.1 h for the oil obtained from whole seeds and 19.1 h for oil from pumpkin seeds.

BCO was characterised by the greatest oxidative stability (38.34 h). Its induction time, compared to the least stable oils, was longer by more than 30 h. In the case of BCO and PO, the high oxidative stability could be due to the high content of natural antioxidants. It should be emphasised that black cumin seed oil was characterised by the highest content of phenolic compounds (Table 1), which contributed to its high resistance to oxidation processes. Many authors, including Monteleone et al. [54] and Morello et al. [55], confirmed the correlation between the phenol content and the oxidative stability of the oil. Due to the origin of the raw material, much lower results were recorded by Qian et al. [21]—14 h and Gharby et al. [56]—13 h, which was positively correlated with the content of oleic acid.

### 3.7. Parameters of the Oxidation Kinetics of Analysed Oils

Determining the parameters of the kinetics of the oil oxidation reaction enables its precise assessment in terms of oxidative stability and suitability for processing [57]. The Rancimat test was performed for each oil at five different temperatures to obtain kinetic parameters. One of the basic parameters of the oxidation reaction kinetics is the activation energy (Ea). This parameter is the smallest amount of energy that a molecule needs to initiate the oxidation reaction. Based on the obtained equations and the Arrhenius equation, the activation energy of the oxidation reaction was calculated for the tested cold-pressed oils. The activation energy of selected cold-pressed oils ranged from 72.23 to 102.02 kJ/mol. Black seed oil, which has the longest induction time (38.34 h), needed the most energy to initiate the oxidation reaction. Equally high results were recorded in the research on olive oil. Ciemniewska-Żytkiewicz et al. [8] for olive oil calculated the activation energy—94.59 kJ/mol, and Gharby et al. [58]—96.28 kJ/mol. High activation energy values characterised the least stable linseed oil (84.99 kJ/mol). The relatively high Ea compared to other oils resulted from the fact that, in the Rancimat method, lower temperatures were used for this oil (90, 95, 100, 105, and 110 °C). According to Farhoosh [59], the temperature is one of the primary factors influencing the parameters of the kinetics of the oxidation reaction. All processes are much slower at lower temperatures, and the molecules need more energy to initiate the oxidation reaction. This assumption is also confirmed by the results of Ratusz et al. [60], which determined the activation energy at the level of 70.39–79.08 kJ/mol, using the following temperatures—80, 90, 100, 110, and 120 °C. HO needed the least energy to initiate the oxidation reaction, despite lower temperatures being used to measure the stability, as in the case of linseed oil. The content of polyunsaturated fatty acids could have influenced such low activation energy; HO had the highest fatty acid content (Table 3). Adhvaryu et al. [61] and Kodali [62] also showed in their works that the activation energy largely depends on the fatty acid composition. Especially PUFAs, which accelerate the oxidation process.

Based on the linear regression equations, the dependence of the logarithm of the oxidation induction time on the reciprocal of the temperature, the Arrhenius equation and the theory of the active complex, the pre-power factor (Z), and the oxidation reaction rate constant (*k*) for each of the measurement temperatures as well as the enthalpy (ΔH) and entropy (ΔS) were calculated. The mentioned parameters of the oxidation reaction kinetics of the tested cold-pressed oils are summarised in Table 5.

The oxidation reaction rate constant (*k*) is directly proportional to the reaction rate. It could be concluded that the reaction rate constant (*k*) increases with increasing temperature. The fastest oxidation rate was recorded for black cumin oil at 120 °C—1.77 h^−1^, and the lowest for black cumin seed oil at 100 °C—0.09 h^−1^. These results correspond to the stability determined in the Rancimat method at 100 °C. Black cumin seed oil with the slowest reaction time had the longest induction time, while oils with a low parameter (*k*), including linseed oil, had low oxidative stability.

Due to the speed of the reaction and low stability, lower temperatures (90–110 °C) were used in HO and LO. The reaction rate constant (*k*) determined for these oils at 90 °C was comparable to the results of the other oils at 100 °C. The value (*k*) for linseed oil was 0.39 h^−1^ (90 °C). Similar results were recorded by Ratusz et al. [60], where the constant reaction rate at 90 °C ranged from 0.31 to 0.39 h^−1^. Kurpiewska [63] determined a much higher reaction rate constant (*k*)—0.74. One of the differences between the results of the constant oxidation rate in linseed oil may be the presence of polyenic acids. The total content of polyunsaturated fatty acids for the tested linseed oil was 75.40% (Table 3), while in the oil tested by Kurpiewska [63] they constituted 67.43% of all the determined acids. In the case of the mixtures of 70% linseed (M1) and 91% linseed (M3), the rates of oxidation at 90 °C were very similar and amounted to 0.35 and 0.37 h^−1^. The Rancimat analysis also showed that these oils do not differ significantly in oxidative stability. RO’s reaction rate constant (*k*) was 0.17 h^−1^ at 100 °C and 0.59 h^−1^ at 120 °C. Slightly higher results for cold-pressed rapeseed oil in their research were recorded by Symoniuk et al. [28]. The rate constant of the oxidation reaction at 100 °C averaged 0.23 h^−1^, and at 120 °C—0.84 h^−1^. The authors also investigated refined rapeseed oil, where the rate constants of the oxidation reaction at 100 °C and 120 °C were 0.06 and 0.34 h^−1^, respectively.

The presence of oxidants in the oil significantly accelerates the oxidation reactions. Cold-pressed oils contain many oxidants in their chemical composition, which are removed during refining. Therefore, cold-pressed oils show much higher rate constants of oxidation according to Kowalski et al. [20]. As part of the analysis of the parameters of the oxidation reaction kinetics of selected cold-pressed oils, the enthalpy (ΔH) was determined, which determined the probability of a spontaneous reaction and the entropy (ΔS), indicating the degree of system disorder and energy dissipation. Both in the case of enthalpy and entropy, BCO had the highest values—98.84 kJ/mol (ΔH) and −70.33 J/mol K (ΔS). Enthalpy and entropy of RO were respectively—73.05 kJ/mol and −133.82 J/mol K, and its mixture (M3)—71.84 kJ/mol and −119.24 J/mol K. LO and its mixtures (M1, M2) had similar enthalpy values from 74.20 to 72.28 kJ/mol. In the case of entropy, LO had a much higher value (−95.06 J/mol K). According to Farhoosh et al. [64], negative entropy values indicate a more significant ordering of active complexes than reactant molecules. Higher entropy values mean a low probability of active complex formation and a slower oxidation reaction. The results obtained in this study confirm this assumption. Furthermore, BCO with the highest parameter value ΔS (−70.33 J/mol K), in the Rancimat apparatus, showed the longest induction time (38.34 h), while the hemp oil with the lowest entropy (−132.93 J/mol K) had one of the shortest induction times (4.32 h).

### 3.8. Influence of Selected Quality Parameters on the Oxidation Stability of Oil

The oxidative stability of cold-pressed oils, due to their rich chemical composition, may depend on many factors. Therefore, it is possible to read the relationships between the individual quality parameters and oxidation stability in the Rancimat at the temperature most commonly used to oxidate cold-pressed oils (100 °C). Principal Component Analysis (PCA) was performed to determine the influence of individual quality parameters on the induction time determined by the Rancimat method. PCA statistical analysis consists in determining two new variables, the so-called major components. A careful principal component analysis allows estimating which variables significantly impact the principal components. The obtained results are presented in the form of a factor load chart.

Table 6 shows the correlation coefficients between the oxidative stability determined at 100 °C and the fifteen selected quality parameters. The PCA analysis included the PV and *p*-AnV, TOTOX index, K232 and K268 coefficients, fatty acid composition, antioxidant capacity, and oxidation kinetic parameters. From the determined parameters of the kinetics of the oxidation reaction, the activation energy values and the constant rate of the oxidation reaction at 100 °C were used for statistical analysis because it is the most frequently used temperature for the oxidation of cold-pressed oils.

The Pearson correlation coefficient distribution table was used to assess the correlation significance. Based on the determined minimum value (0.51), the significance of individual correlations was estimated. The value of 0.51 was read based on the degree of freedom and the significance level of 0.05. The obtained correlation coefficient above the value of 0.51 read from the Pearson correlation coefficient distribution table indicated a statistically significant relationship between a given quality parameter and the oxidative stability determined in the Rancimat test. In contrast, the results below this value showed no significant correlation. Based on the data in Table 6, it can be concluded that the oxidative stability of the tested oils depends on many quality parameters. The greatest influence on the oxidation stability was exerted by the PV and TOTOX; correlation with stability was r = 0.89. The kinetic parameters were also important, including the activation energy (r = 0.77), the rate constant of the oxidation reaction (k at 100 °C) (r = −0.75), the content of primary and secondary oxidation products (r = 0.71, r = 0.72), the total content of phenolic compounds (r = 0.69), and the ability to reduce DPPH free radicals (r = −0.58). The *p*-AnV and antioxidant capacity determined using ABTS cation radicals had no significant effect on the oxidative stability of the tested cold-pressed oils. The determined correlation coefficients for these parameters were very similar and amounted to 0.43 and 0.42, respectively. The performed statistical analysis showed that the overall content of individual fatty acid groups, including SFA (r = 0.48), PUFA (r = −0.30), and MUFA (r = 0.19), also did not have a significant effect on the induction time, determined by the Rancimat method, of oil oxidation at 100 °C. From the parameters of fatty acid composition, only the content of α-linolenic acid (C18:3) significantly decreased the oxidative stability of the oils (r = −0.64).

Ratusz et al. [65] also noted the strongest correlation between the oxidative stability and the PV (r = 0.86) and the TOTOX index calculated on its basis (r = 0.83) in the PCA analysis of the oxidative stability of camelina oil. On the other hand, Symoniuk et al. [4] showed that the content of chlorophyll and carotenoids determined by them had the most significant impact on the stability of the different cold-pressed oils (r = 0.66). In the studies by Symoniuk et al. [4], no significant effect of fatty acid composition on oxidative stability was found. The correlation coefficient for the dependence of oxidative stability on the content of SFA, MUFA, PUFA was, respectively, 0.20, −0.11, −0.05. The correlations between the oxidative stability determined by the Rancimat method at 100 °C and the individual quality parameters were verified by determining the main components with PCA statistical analysis. Table 7 presents the main component (PCA) values for fifteen selected quality parameters, the two main factors (PC1 and PC2), and their values for the chosen quality parameters of the tested cold-pressed oils. PC1 was mainly caused by the TOTOX indicator (0.90), peroxide value (0.89), and induction time (0.90), while the total content of polyunsaturated (0.95) and monounsaturated (0.90) fatty acids contributed significantly to PC2.

**Figure 1:**
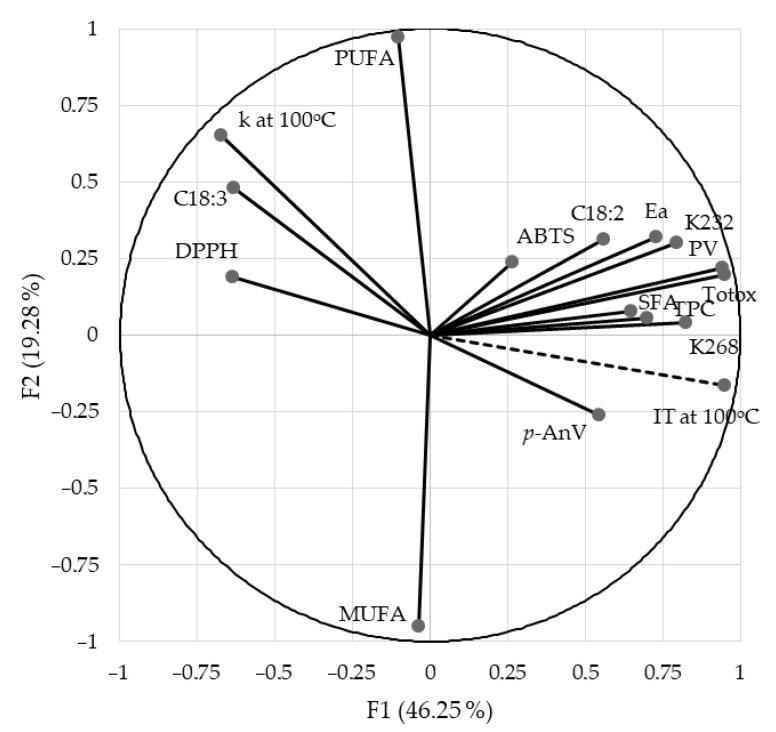
Loading and score plots of PC1–PC2 for different cold-pressed oils and their mixtures divided into oxidation stability assessment method.

Principal Component Analysis (PCA) extracts and stores the most important data. PCA creates linear combinations of principal components that describe the variability between individual features. Figure 1 presents a graphical arrangement of selected quality characteristics to the oxidation stability of oils, determined in the Rancimat test at 100 °C. The main factors accounted for 65.53% of the variation (PC1: 19.28 and PC2: 46.25%). The analysis of the main components illustrates the correlation of the discussed quality attributes with regard to oxidative stability.

Principal component analysis (PCA) confirmed the strongest significant positive correlation between oxidative stability and the peroxide value and TOTOX index. As shown in Figure 1, the activation energy, the content of conjugated dienes and trienes, and the presence of phenolic compounds also significantly impact the oxidation induction time. A significant correlation between the oxidative stability and individual parameters was also noted by Papadimitriou et al. [66], Kruszewski et al. [27], and Symoniuk et al. [4]. The obtuse angle between the stability vector and the α-linolenic acid content vector (C18:3) and the rate constant of the oxidation reaction at 100 °C (*k* at 100 °C) indicates a significant negative correlation between these features. Neđeral et al. [10] also confirmed that the oxidative stability of the oil is negatively correlated with the content of polyunsaturated α-linolenic acid.

The graph of principal component analysis (PCA) shows the influence of individual parameters on oxidative stability and the correlations between these parameters. Figure 1 shows that the activation energy and the content of conjugated dienes are positively correlated, as is the constant of the oxidation reaction rate at 100 °C and the content of α-linoleic acid. There is a negative correlation between the total content of polyphenols and the ability to scavenge DPPH free radicals. This relationship was also confirmed in the studies by Siger et al. [40].

**Figure 2:**
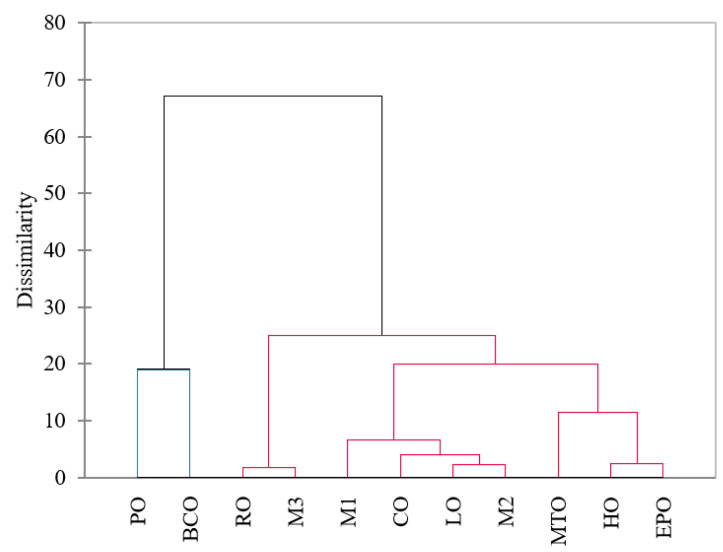
Agglomerative Hierarchical Clustering (AHC) for analysed oils.

The dendrogram resulting from cluster analysis ordered quality parameters of oils in a hierarchical manner, applying the nearest neighbour method with the Euclidean distance measure based on presence or absence of a particular component. In our study, oil samples were classified by the different quality parameters. Figure 2 shows the dendrogram obtained from HCA, where two clusters can be identified. Cluster 1 contains PO and BCO and cluster 2 contains the other oils (HO, LO, CO, RO, MTO, EPO, M1, M2, and M3).

## 4. Conclusions

Due to the specificity of the raw materials and different use-by dates, the tested cold-pressed oils differed in terms of basic physicochemical parameters. Cold-pressed oils were characterised by a relatively high content of unsaturated fatty acids. The best source of n-3 family α-linolenic acid was linseed oil (53.90%), which can be used as an additive in other oils to improve the n-6 to n-3 ratio. However, the high content of this acid has a negative impact on the oxidation stability; therefore, LO was characterised by the shortest induction time among all the tested oils. The composition of fatty acids, including the total content of saturated, monounsaturated, and polyunsaturated fatty acids, had no significant influence on the oxidative stability of the tested cold-pressed oils. The α-linolenic fatty acid showed a significant correlation with the induction time determined in the Rancimat method at 100 °C. The presence of this acid significantly decreased the resistance of oils to the oxidation processes. The highest content of α-linolenic acid was found in LO (53.90%), and the lowest in BCO (0.07%). The presence of phenolic compounds in the oil significantly impacted their oxidation stability. BCO was the most resistant to the oxidation processes. PO and RO also had relatively good antioxidant resistance. The shortest induction time was determined for LO, its mixtures (M1,M2), and HO. In the case of linseed mixtures, the addition of other oils, including EPO and MTO, did not significantly increase their resistance to the oxidation processes. The Rancimat method confirmed the close relationship between oil stability and the process temperature. The activation energy, based on the analysis of the oxidation induction times, showed a high correlation with the stability of oils. The high-energy value needed by a molecule to initiate an oxidation reaction means a long induction time and high oxidation stability. BCO (102.02 kJ/mol) needed the most energy to initiate oxidation among the analysed cold-pressed oils. Many factors influence the oxidative stability of cold-pressed oils in the Rancimat method at 100 °C. The most important is the PV and the TOTOX index calculated on its basis, activation energy, and the K232 and K268 coefficients. A significant correlation with the induction time was also shown by the reaction rate constant (*k*) and the content of α-linolenic acid. The composition of fatty acids and the level of secondary oxidation products defined as the anisidine number slightly influenced the oxidative stability of all the tested oils.

## Figures and Tables

**Table 1:** Initial quality of analysed oils.

Oil	PV	*p*-AnV	TOTOX	K232	K268
PO	42.52 ^d^	7.14 ^g^	98.96 ^e^	4.57 ^h^	3.63 ^i^
HO	19.42 ^c^	2.68 ^d^	41.51 ^d^	2.10 ^e^	0.50 ^g^
LO	0.95 ^a^	0.49 ^a^	2.38 ^a^	1.86 ^cd^	0.31 ^e^
CO	4.91 ^b^	1.92 ^c^	11.74 ^bc^	1.75 ^c^	0.26 ^c^^,^^d^
RO	0.85 ^a^	3.55 ^e^	5.25 ^a^	1.45 ^b^	0.17 ^b^
BCO	81.93 ^e^	3.91 ^e^	167.76 ^f^	5.61 ^i^	2.20 ^h^
MTO	0.92 ^a^	1.14 ^b^	2.98 ^a^	0.26 ^a^	0.03 ^a^
EPO	4.70 ^b^	1.16 ^b^	10.56 ^b^	2.85 ^g^	0.28 ^d,e^
M1	0.33 ^a^	5.51 ^f^	6.16 ^a^	2.49 ^f^	0.27 ^d,e^
M2	5.83 ^b^	2.76 ^d^	14.42 ^c^	2.80 ^g^	0.41 ^f^
M3	0.35 ^a^	2.59 ^d^	3.29 ^a^	1.94 ^d^	0.23 ^c^

^a–i^—Different letters in the column indicate significant difference between values (*p* < 0.05). PV—peroxide value; *p*-AnV—*p*-anisidine value; Totox—total oxidation indictor; K232 and K268—specific extinction coefficient at wavelength λ = 232 and 268. PO—pumpkin oil; HO—hemp oil; LO—linseed oil; CO—camelina oil; RO—rapeseed oil; BCO—black cumin oil; MTO—milk thistle oil; EPO—evening primrose oil; M1—70% LO, 27% MTO, 3% EPO; M2—80% RO, 17% LO, 3% EPO; M3 –91% LO, 5% EPO, 4% PO.

**Table 2:** Fatty acid composition of analysed oils.

Fatty Acid						Oil					
	PO	HO	LO	CO	RO	BCO	MTO	EPO	M1	M2	M3
C16:0	12.23	5.79	5.43	5.51	4.59	10.98	8.51	7.30	6.19	6.06	6.11
C18:0	5.55	2.56	3.17	2.48	1.74	2.62	5.10	2.55	4.47	3.02	1.34
C18:1	30.21	12.71	14.45	16.42	70.28	24.45	29.54	16.12	25.18	14.34	56.07
C18:2	51.20	59.41	21.36	19.33	17.07	60.12	53.03	71.53	25.9	20.46	17.43
C18:3	0.21	17.95	53.90	37.92	5.30	0.07	0.22	1.80	37.30	56.03	18.02
C20:0	0.34	0.83	-	1.35	-	0.04	1.90	-	0.08	-	-
C20:1	0.11	0.75	1.13	15.56	1.02	0.13	-	0.70	0.49	0.09	1.03
C20:2	-	-	0.14	1.43	-	1.59	-	-	-	-	-
C22:0	0.15	-	0.42	-	-	-	1.70	-	0.39	-	-
∑SFA	18.27	9.18	9.02	9.34	6.33	13.64	17.21	9.85	11.13	9.08	7.45
∑MUFA	30.32	13.46	15.58	31.98	71.30	24.58	29.54	16.82	25.67	14.43	57.10
∑PUFA	51.41	77.36	75.40	58.68	22.37	61.78	53.25	73.33	63.2	76.49	35.45

SFA—saturated fatty acids; MUFA—monounsaturated fatty acids; PUFA—polyunsaturated fatty acids. PO—pumpkin oil; HO—hemp oil; LO—linseed oil; CO—camelina oil; RO—rapeseed oil; BCO—black cumin oil; MTO—milk thistle oil; EPO—evening primrose oil; M1—70% LO, 27% MTO, 3% EPO; M2—80% RO, 17% LO, 3% EPO; M3—91% LO, 5% EPO, 4% PO.

**Table 3:** Antioxidant activity of analysed oils.

Oil	DPPH			ABTS			TPC [mg GAE/100 mg]
	AA [mM TEAC/kg]	% Inhibition	TEC_50_ [min]	AA [mM TEAC/kg]	% Inhibition	TEC_50_ [min]	
PO	2.54 ^a,b^	50.07 ^b^	57.5 ^g^	4.19 ^b^	38.83 ^b^	-	110.92 ^c^
HO	3.53 ^f,g^	83.62 ^c^	5.77 ^d^	5.49 ^d^	48.71 ^e^	12.99 ^c^	236.25 ^f^
LO	3.45 ^e,f^	82.83 ^c^	5.21 ^c^	8.43 ^h^	68.29 ^i^	0.92 ^a^	56.61 ^a^
CO	3.35 ^e^	77.46 ^i^	3.66 ^b^	9.46 ^i^	72.35 ^j^	2.48 ^a^	132.67 ^d^
RO	3.12 ^d^	69.86 ^h^	7.01 ^e^	7.53 ^g^	62.46 ^h^	1.00 ^a^	162.43 ^e^
BCO	2.41 ^a^	44.69 ^a^	-	11.60 ^j^	92.55 ^k^	0.05 ^a^	384.66 ^h^
MTO	2.59 ^b^	50.98 ^d^	49.77 ^f^	3.87 ^a^	36.53 ^a^	-	252.87 ^g^
EPO	3.65 ^g^	88.47 ^j^	1.85 ^a^	5.29 ^c^	47.86 ^d^	26.99 ^d^	128.77 ^d^
M1	2.51 ^a,b^	49.93 ^b^	-	5.23 ^c^	45.85 ^c^	-	125.27 ^d^
M2	2.91 ^c^	62.52 ^f^	7.07 ^e^	6.34 ^f^	54.01 ^g^	1.78 ^a^	78.26 ^b^
M3	3.09 ^c,d^	68.81 ^g^	5.35 ^d^	5.73 ^e^	49.14 ^f^	7.82 ^b^	87.53 ^b^

^a–k^—Different letters in the column indicate significant difference between values (*p* < 0.05). AA—antioxidants activity; TEC_50_—time needed to reduce 50% of DPPH or ABTS radicals; TPC—total phenolic content; TEAC—trolox equivalent; GDA—gallic acid equivalents. PO—pumpkin oil; HO—hemp oil; LO—linseed oil; CO—camelina oil; RO—rapeseed oil; BCO—black cumin oil; MTO—milk thistle oil; EPO—evening primrose oil; M1—70% LO, 27% MTO, 3% EPO; M2—80% RO, 17% LO, 3% EPO; M3—91% LO, 5% EPO, 4% PO.

**Table 4:** Induction time of analysed oils at temperatures from 80 to 120 °C.

Oil	Induction Time [h]						
	80 °C	90 °C	100 °C	105 °C	110 °C	110 °C	120 °C
PO	-	-	22.45 ± 0.05	17.04 ± 0.05	13.01 ± 0.16	8.62 ± 0.01	5.67 ± 0.05
HO	8.45 ± 0.08	6.52 ± 0.06	4.32 ± 0.02	3.65 ± 0.03	2.26 ± 0.06	-	-
LO	7.11 ± 0.02	5.73 ± 0.07	3.37 ± 0.14	2.66 ± 0.05	1.55 ± 0.01	-	-
CO	-	-	4.62 ± 0.05	3.57 ± 0.05	2.69 ± 0.06	1.89 ± 0.04	1.29 ± 0.03
RO	-	-	15.45 ± 0.06	10.99 ± 0.01	8.00 ± 0.10	5.77 ± 0.11	4.22 ± 0.04
BCO	-	-	38.34 ± 0.15	29.00 ± 0.06	18.98 ± 0.07	10.71 ± 0.12	7.12 ± 0.13
MTO	-	-	11.17 ± 0.02	8.21 ± 0.04	6.23 ± 0.05	4.23 ± 0.03	2.66 ± 0.02
EPO	-	-	7.20 ± 0.16	4.94 ± 0.18	3.65 ± 0.01	2.55 ± 0.12	1.85 ± 0.06
M1	7.30 ± 0.01	5.99 ± 0.04	3.63 ± 0.09	2.94 ± 0.01	1.84 ± 0.04	-	-
M2	7.14 ± 0.02	5.83 ± 0.07	3.47 ± 0.06	2.78 ± 0.01	1.68 ± 0.01	-	-
M3	-	-	8.68 ± 0.01	6.91 ± 0.05	5.02 ± 0.04	4.67 ± 0.04	3.68 ± 0.11

PO—pumpkin oil; HO—hemp oil; LO—linseed oil; CO—camelina oil; RO—rapeseed oil; BCO—black cumin oil; MTO—milk thistle oil; EPO—evening primrose oil; M1—70% LO, 27% MTO, 3% EPO; M2—80% RO, 17% LO, 3% EPO; M3—91% LO, 5% EPO, 4% PO.

**Table 5:** Oxidation kinetics parameters of analysed oils.

Oil	Z	Kinetic Parameter									
		k [h^−1^]							Ea [kJ/mol]	ΔH [kJ/mol]	ΔS [J/mol K]
		80 °C	90 °C	100 °C	105 °C	110 °C	115 °C	120 °C			
PO	2.07 × 10^10^	-	-	0.11	0.16	0.23	0.31	0.43	80.40	77.22	−126.02
HO	7.16 × 10^9^	0.29	0.40	0.55	0.75	1.01	-	-	72.23	69.12	−132.93
LO	6.55 × 10^11^	0.39	0.57	0.83	1.19	1.70	-	-	84.99	81.87	−95.06
CO	1.18 × 10^10^	-	-	0.53	0.72	0.98	1.32	1.77	73.94	70.76	−130.56
RO	7.99 × 10^9^	-	-	0.17	0.24	0.32	0.44	0.59	76.23	73.05	−133.82
BCO	1.65 × 10^13^	-	-	0.09	0.13	0.20	0.31	0.46	102.02	98.84	−70.33
MTO	1.04 × 10^11^	-	-	0.24	0.34	0.48	0.68	0.94	83.11	79.93	−119.71
EPO	4.26 × 10^10^	-	-	0.38	0.53	0.74	1.01	1.38	78.94	75.76	−123.41
M1	4.61 × 10^10^	0.35	0.49	0.69	0.96	1.32	-	-	77.32	74.20	−123.97
M2	1.36 × 10^11^	0.37	0.53	0.76	1.06	1.48	-	-	80.39	77.28	−104.30
M3	5.83 × 10^9^	-	-	0.18	0.25	0.34	0.47	0.62	75.03	71.84	−119.24

Ea—activation energy; ΔH—entalphy; ΔS—entropy; k—reaction rate coefficient; Z—pre-exponential factor; PO—pumpkin oil; HO—hemp oil; LO—linseed oil; CO—camelina oil; RO—rapeseed oil; BCO—black cumin oil; MTO—milk thistle oil; EPO—evening primrose oil; M1—70% LO, 27% MTO, 3% EPO; M2—80% RO, 17% LO, 3% EPO; M3—91% LO, 5% EPO, 4% PO.

**Table 6:** Correlation coefficient between oil chemical component and oxidative stability in Rancimat.

Nr	Parameter	IT at 100 °C [h]
1	PV	**0.89**
2	*p*-AnV	0.43
3	TOTOX	**0.89**
4	K_232_	**0.71**
5	K_268_	**0.72**
6	MUFA	0.19
7	PUFA	−0.30
8	SFA	0.48
9	C18:2	0.40
10	C18:3	**−0.64**
11	TPC	**0.69**
12	DPPH	−0.58
13	ABTS	0.42
14	Ea	**0.77**
15	*k* at 100 °C	**−0.75**

The values in bold show the correlations statistically significant at the significance level *p* = 0.05. PV—peroxide value; *p*-AnV—*p*-anisidine value; Totox—total oxidation indictor; K232 and K268—specific extinction coefficient at wavelength λ = 232 and 268. MUFA—monounsaturated fatty acids; PUFA—polyunsaturated fatty acids; SFA—saturated fatty acids; TPC—total phenolic content; DPPH—antioxidants activity measured using DPPH radicals; ABTS—antioxidants activity measured using ABTS radicals; Ea—activation energy; *k*—reaction rate coefficient.

**Table 7:** Principal component analysis (PCA) factor loadings for the quality factors of analysed oils.

Parameter	PC1	PC2
PV	**0.89**	0.05
AnV	0.30	0.07
TOTOX	**0.90**	0.04
K_232_	**0.63**	0.09
K_268_	**0.68**	0.00
MUFA	0.01	**0.90**
PUFA	0.01	**0.95**
SFA	**0.42**	0.01
C18:2	**0.31**	0.10
C18:3	**0.40**	0.23
TPC	**0.49**	0.00
DPPH	**0.41**	0.04
ABTS	0.07	0.06
Ea	**0.53**	0.10
*k* at 100 °C	**0.45**	0.43
IT at 100 °C	**0.90**	0.03

Values in bold correspond for each variable to the factor for which the squared cosine is the largest. PV—peroxide value; *p*-AnV—*p*-anisidine value; TOTOX—total oxidation indictor; K232 and K268—specific extinction coefficient at wavelength λ = 232 and 268. MUFA—monounsaturated fatty acids; PUFA—polyunsaturated fatty acids; SFA—saturated fatty acids; TPC—total phenolic content; DPPH—antioxidants activity measured using DPPH radicals; ABTS—antioxidants activity measured using ABTS radicals; Ea—activation energy; *k*—reaction rate coefficient; IT—induction time.

## Data Availability

The data presented in this study are available on request from the corresponding author.
